# Effect of local application of bone morphogenetic protein -2 on experimental tooth movement and biological remodeling in rats

**DOI:** 10.3389/fphys.2023.1111857

**Published:** 2023-04-18

**Authors:** Menglin Wang, Jiadong Fan, Aoao Wang, Xiang Jin, Zhenbao Zhang, Xiantong Hu, Le Liu, Yantao Zhao, Yanfeng Li

**Affiliations:** ^1^ Medical School of Chinese PLA, Beijing, China; ^2^ Department of Stomatology, The Fourth Medical Centre, Chinese PLA General Hospital, Beijing, China; ^3^ Senior Department of Orthopedics, The Fourth Medical Centre, Chinese PLA General Hospital, Beijing, China; ^4^ Beijing Engineering Research Center of Orthopedics Implants, Beijing, China; ^5^ State Key Laboratory of Military Stomatology, Xi’an, China

**Keywords:** orthodontics, bone remodeling, osteoclastogenesis, osteogenesis, anchorage, local delivery

## Abstract

**Background:** This study attempts to detect the potential effects of local bone morphogenetic protein -2 (BMP-2) on orthodontic tooth movement and periodontal tissue remodeling.

**Methods:** Forty adult SD rats were randomly divided into four groups: blank control group, unilateral injection of BMP-2 on the pressure side or tension side of orthodontic teeth and bilateral injection of BMP-2. Their maxillary first molar was moved by a 30 g constant force closed coil spring. 60 μL of BMP-2 with a concentration of 0.5 μg/mL was injected into each part at a time. In addition, three rats were selected as healthy control rats without any intervention. Fluorescent labeled BMP-2 was used to observe the distribution of exogenous BMP-2 in tissues. Micro-CT was used to measure the microscopic parameters of tooth displacement, trabecular bone and root absorption volume. Three different histological methods were used to observe the changes of tissue remodeling, and then the number of osteoclasts and the content of collagen fibers were calculated.

**Results:** Compared with the blank control group, BMP-2 injection reduced the movement distance and increased the collagen fiber content and bone mass (*p* < 0.01). There was no significant difference in tooth movement distance, BV/TV ratio and BMD between injection sites in unilateral injection group (*p* > 0.05). In the case of bilateral injection of BMP-2, the osteogenesis is enhanced. Unilateral injection of BMP-2 did not promote root resorption, but double injection showed root resorption (*p* < 0.01).

**Conclusion:** Our study does show that the osteogenesis of BMP-2 is dose-dependent rather than site-dependent when a certain amount of BMP-2 is applied around orthodontic teeth. Local application of BMP-2 around orthodontic teeth in an appropriate way can enhance bone mass and tooth anchorage without increasing the risk of root absorption volume. However, high levels of BMP-2 may cause aggressive root resorption. These findings are of great significance, that is, BMP-2 is an effective target for regulating orthodontic tooth movement.

## Introduction

By applying spatial analysis and more adjustable movement, orthodontic goals can be achieved more accurately and effectively. Orthodontic tooth movement (OTM) is achieved through the remodeling of periodontal ligament (PDL) and alveolar bone, in response to controlled mechanical load, bone absorption under pressure and bone formation under tension (Melsen, 2001). Bone remodeling is considered to be the decisive factor of orthodontic tooth movement, and osteoblasts and osteoclasts are the main cell effectors (Baloul et al., 2011).

Efficient orthodontic augmentation devices, osteoprotegerin (OPG) and sclerostin as potential targets, physical therapy techniques (such as low laser therapy and vibration), surgical treatment and gene therapy are all effective methods to regulate OTM (Fernández-González et al., 2016; Sugimori et al., 2018; Isola et al., 2019; Lu et al., 2019; Sakamoto et al., 2019; Sivarajan et al., 2019; Yoo-Sung et al., 2022). Bone reconstruction is a key component of most methods. There are several cytokines and signal molecules that affect the biological trend and rate of bone remodeling, which may be related to tooth movement in theory (Lee et al., 2021; Li et al., 2021). Bone morphogenetic protein-2 (BMP-2) is essential for controlling the proliferation, differentiation and secretion of bone matrix of osteoblasts, and regulating osteoclast differentiation and activating bone resorption through RANKL/OPG pathway (Kamiya and Mishina, 2011). The level of BMP-2 is related to the histological changes during OTM, and the expression of BMP-2 can be used as a marker to detect osteogenic activity and evaluate the efficacy of various techniques to regulate bone remodeling (Kamiya and Mishina, 2011). Due to mechanical contraction, BMP-2 protein level and gene expression on the tension side can be significantly enhanced (Wescott et al., 2007).

In the dental application of bone growth, the concentration of BMP-2 ranges from 0.5 μg/mL to 2.5 μg/mL (Stenport et al., 2003; Vercellotti and Podesta, 2007; Razzouk and Sarkis, 2012). It is reported that the combined application of BMP-2 and periodontal accelerated osteogenesis orthodontics (PAOO) can shorten the treatment time and increase bone mineral density compared with traditional cortical resection (Gao et al., 2021). However, this is still an ongoing debate, because some authors have found that the application of BMP-2 on the tension side can not promote OTM acceleration (Iglesias-Linares et al., 2012; Kurohama et al., 2017). The role of exogenous BMP-2 in the regulation of tooth mobility or bone remodeling of orthodontic teeth has not been clarified. There is still uncertainty about whether local application of BMP-2 around orthodontic teeth can enhance the resistance to traction, or on the contrary, whether it can promote the movement rate by accelerating the pace of bone reconstruction. The purpose of this study is to evaluate the overall effect of clinically acceptable dose of BMP-2 on orthodontic tooth mobility and biological tissue remodeling.

## Materials and methods

### Orthodontic tooth movement model

**FIGURE 1:**
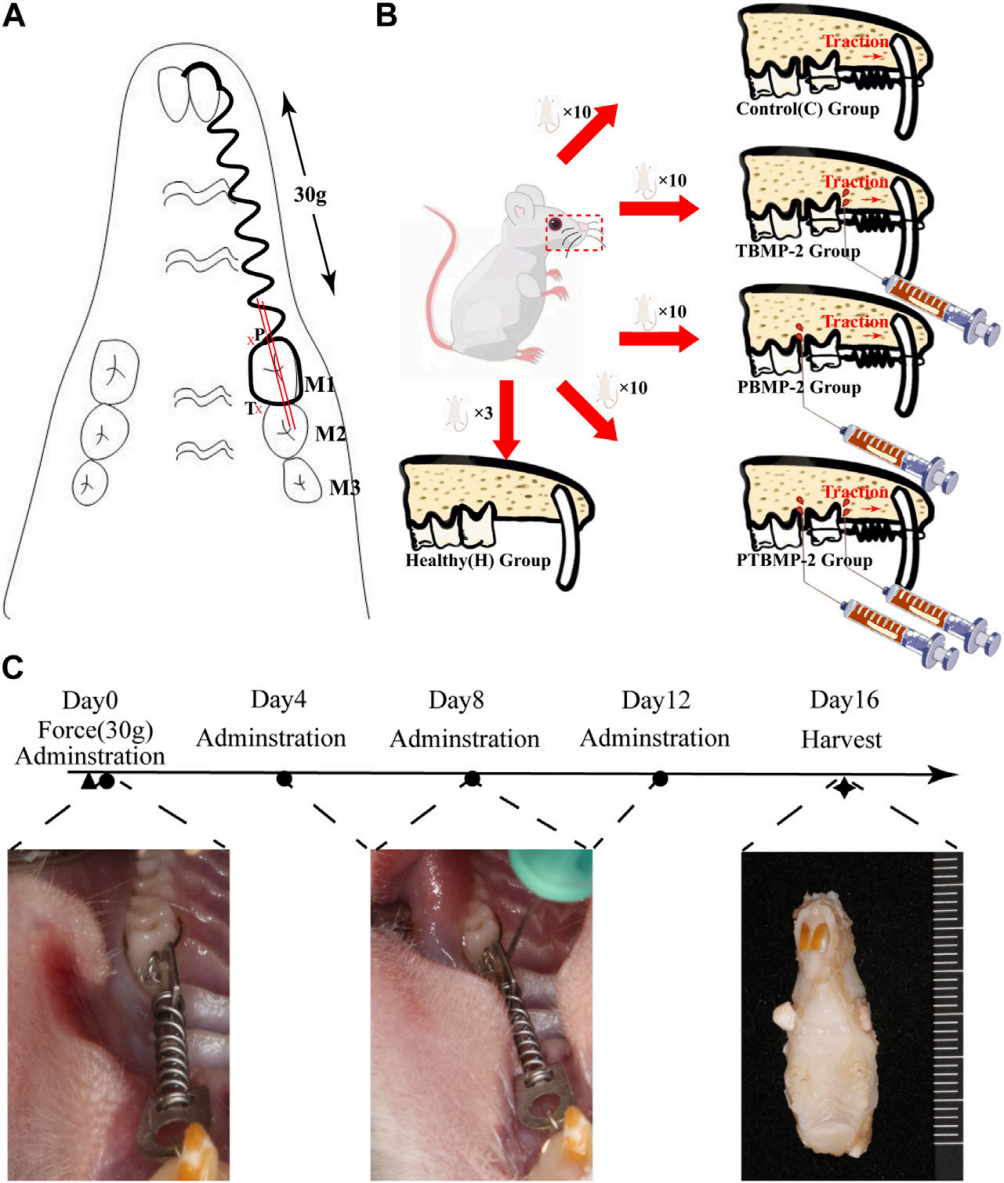
Experimental design. Establishment of orthodontic tooth movement (OTM) model **(A)**. A closed coil NiTi spring was ligated between the left maxillary central incisor and the first molar (M1). A constant force of 30 g was ensured. The administration site was defined on the pressure side (P) and tension side (T) side of the orthodontic tooth 0.5 mm close to the periodontal ligament. The whole morphology of the mesial root of M1 was captured in sections taken from the mesial and distal sides (double red bar) along the long axis of M1. Group distribution **(B)**. 40 healthy SD rats were randomly allocated into four groups based on different interventions on the established OTM model: 1-blank control **(C)** group without intervention on the orthodontic tooth; 2-Pressure BMP-2(PBMP-2) group with administration of BMP-2 on the pressure side of the orthodontic tooth; 3-Tension BMP-2 (TBMP-2) group with administration of BMP-2 on the tension side of the orthodontic tooth; 4-Pressure and Tension BMP-2 (PTBMP-2) group with administration of BMP-2 on both the pressure and tension side of the orthodontic tooth. Three healthy SD rats were selected and housed in the identical condition to the healthy control (H) group without any intervention. The flow of the experiment contains the time point of intervention and intraoral representation of the experimental procedures **(C)**. The first injection was administered immediately after the placement of the orthodontic augmentation devices (Day 0), and then every 3 days (Day 4, Day 8, and Day 12). All samples were sacrificed after 16 days. ◀ Represents the installation of the orthodontic augmentation devices, ● represents the injection of BMP-2, ★ represents the execution of animals for material.

The rat OTM model used in previous studies was established (King et al., 1991; Zhou et al., 2019). Rats were anesthetized by intraperitoneal injection of 10% ketamine hydrochloride (0.08 mL per 100 g body weight) and 2% tolthiazine hydrochloride (0.04 mL per 100 g body weight). Using a dental diamond drill, the retention notches were ground on the distal, mesial and labial sides of the left maxillary incisors and the mesial and buccal and lingual sides of the left maxillary first molar (M1). The incisors and the neck of M1 were ligated with 0.2 mm ligature (CrNi, Morelj, Brazil), and the tooth surface was etched with 35% phosphoric acid (Gluma, Etch 35%, Heraeus Kulzer, Germany) for 30 s, washed, dried, insulated, and coated with adhesive (Transbond, 3M Unitek, Monrovia, California). A thin layer of resin (Filtek Flow composite, 3M/ESPE, St. Paul, MN, USA) was covered on the surface of the line and incision to prevent the dislocation of the ligature line. A closed coil NiTi spring (Light, Sentalloy, GAC, Dentsply) was ligated between the left maxillary central incisor and M1 for traction (Figure 1A), and the force was calculated by an dynamometer (YS-31D, YDM). Check this force activation device every day, and if it is damaged or released, immediately reinstall or add rat specimens.

### Experimental grouping

All animal studies were conducted in accordance with the guidelines of the National Institutes of Health (NIH) and approved by the Fourth Medical Center of the General Hospital of the Chinese People’s Liberation Army (approval number ZJU20160455). The animals included in this study adapted to the environment for at least 1 week before the experiment. Forty Sprague-Dawley rats (8 weeks old, 280–300 g) were randomly divided into four groups according to different administration procedures (Figure 1B):1- blank control group (C) without any intervention on orthodontic teeth; In 2- pressure BMP-2 (PBMP-2) group, BMP-2 was applied to the pressure side of orthodontic teeth; In 3- tension BMP-2 (TBMP-2) group, BMP-2 was applied to the tension side of orthodontic teeth; In the 4- pressure and tension BMP-2 (PTBMP-2) group, BMP-2 was applied on the pressure and tension sides of orthodontic teeth. The other three SD rats were placed under the same conditions as the healthy control group (H) without any intervention.

### BMP-2 injection

The recombinant protein BMP-2 (molecular weight 13 kDa, R&D Systems, Minneapolis, Minnesota) was recombined according to the manufacturer’s instructions. Freeze-dried from 0.2 micron filtered solution in glycine, sucrose, Tween 80 and glutamic acid, using BSA as carrier protein. * Provide 1 mg package size (01 M) in the form of 0.2 m filtered solution in glycine, sucrose, Tween 80 and glutamic acid, with BSA as carrier protein. Reconstructed in sterile 4 mM HCl containing at least 0.1% human or bovine serum albumin at a concentration of 100–200 μg/mL. And adjust to that concentration of 0.5 μg/mL/60 μL injection to produce BMP-2. BMP-2 was injected with Hamilton micro syringe (microliter syringe) within 0.5 mm near periodontal ligament on the pressure or tension side of orthodontic teeth (Figure 1A). The first injection was made immediately after the orthodontic appliance was installed (day 0), and then the injection was made with ether anesthetic every 3 days (day 4, day 8 and day 12). After 16 days, all the samples were killed due to an overdose of narcotic drugs (Figure 1C). During this period, it has been proved that significant tooth movement has been achieved (Marcantonio et al., 2021; Nogueira et al., 2017). After death, the left maxilla of each sample was taken out, and then reset and randomly assigned for micro-computed tomography (micro-CT) and histological analysis.

### Distribution of exogenous BMP-2 in periodontal tissues of OTM rats

Three samples were randomly selected from each experimental BMP-2 injection group and given fluorescently labeled BMP-2 (R&D Systems, Minneapolis, Minnesota). One sample in each group was killed on the first, second, and third day after injection. After decalcification and dehydration, the left maxillary samples were collected and sliced. The distribution of BMP-2 in periodontal tissue was observed by confocal laser scanning microscope (Olympus, Japan). Microscopic CT examination.

**FIGURE 2:**
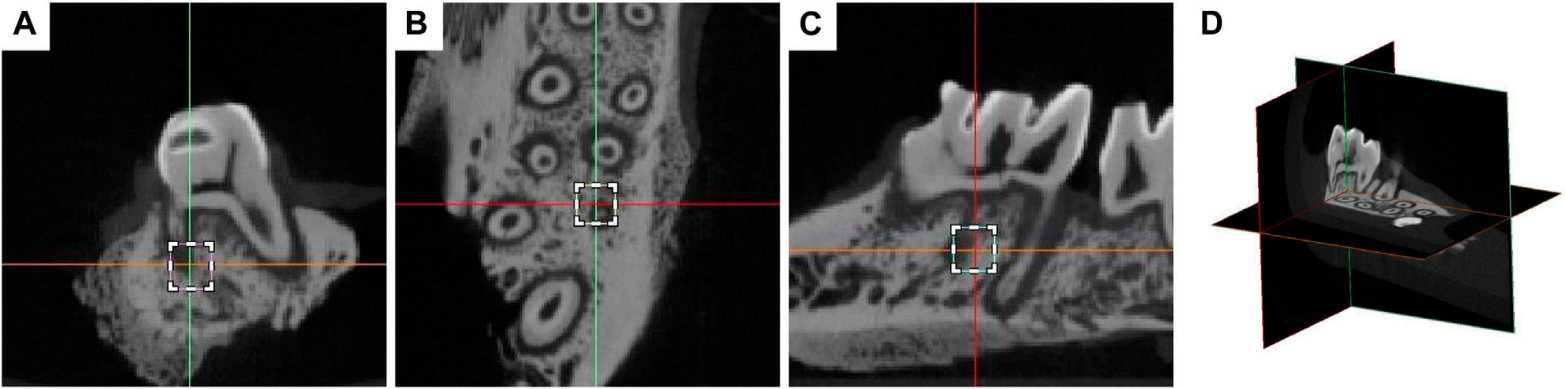
Definition of the region of interest (ROI) on the coronal view **(A)**, axial view **(B)**, sagittal view **(C)** of micro-CT, and the three-dimensional overall view **(D)**. The region of ROI was defined as a 700 μm × 700 μm × 700 μm cube in the mesial alveolar bone interval in the middle 1/3 of the distal and labial root, 200 μm from the root surface.

After the rats were killed, the left maxilla was taken out, and the repaired bone was immediately placed on the scanning table with a micro-CT machine (Skyscan 1176 Bruker, Kontich, Belgium). The scanning parameters were set to 70 kVp voltage, 114 uA current, 8 W, 38.9 mm viewing angle diameter and 10 μm image resolution. Using nrecon (Bruker version 1.6.1.5), Data Viewer (Bruker version 1.4.3.1) and CTAN (Bruker version 1.10.1.0 by CTan (CT-Analyser software), the 3D image was reconstructed, and the image location and stereological analysis were carried out. The region of interest (ROI) is defined as a cube of 700 μm × 700 μm × 700 μm, which is located in the mesial alveolar bone space between the distal end and the labial root, and is 200 μm away from the root surface (Figure 2). Root and periodontal ligament were excluded from ROI. The microstructure parameters evaluated included bone volume fraction (BV/TV), bone mineral density (BMD) and trabecular number (Tb). N), trabecular separation (Tb. Sp) and trabecular thickness (Tb. Th). The volume of the absorption pit (106 μm^3^) on the mesial root of M1 cementum surface was measured in Mimics Medical 21.0 software (Materialise, Belgium), and the convex hull algorithm was used for calculation. In order to determine the tooth movement, the horizontal distance between the distal surface of M1 and the highest convex surface of the proximal surface of M2 was measured (Nogueira et al., 2021).

### Histological analysis

The left half of the maxilla of each sample was fixed in 4% formaldehyde with pH7.2 buffered with 0.1 M sodium phosphate for 24 h, and then decalcified in EDTA (EDTA 10%, 0.5 M) at 4°C for 6–8 weeks until the needle tip could pierce the teeth without resistance, and then embedded in paraffin for subsequent tissue sections. A series of 4 μm parasagittal sections were fixed on a glass slide. Cut the slice from proximal to distal along the long axis of the tooth. For each staining method, 10 slices containing the complete morphology of the proximal root of M1 were selected from each sample.

HEmatoxylin-eosin (He) staining was used to observe the histological changes. Masson staining was performed to observe collagen fibers. We also measured the contents of collagen fibers on the pressure side and the tension side of M1 in each standard visual field of the selected slice. The volume fraction (CVF) of collagen was calculated by ImageJ (ImageJ 1.53b, National Institutes of Health). Tartaric acid phosphatase (TRAP) staining was used to observe osteoclasts and cementum-destroying cells. Three standard visual fields, the neck, the middle third of the root and the apical area, were selected on each side and observed under 40 times magnification. The number of red stained nucleated cells in every three standard visual fields was calculated for each slice and averaged. All analyses were performed by calibrated blind reviewers. The same inspector measures each part twice.

Samples of organs (heart, liver, spleen, lung and kidney) of rats were collected after execution. All the collected organ samples were fixed in 10% neutral formaldehyde fixative, dehydrated, embedded and stained with HE, and the possible pathological changes of organs were observed.

### Statistical analysis

SPSS 23.0 software (IBM, Chicago, il) was used for statistical analysis. Shapiro-wilk test and D’Agostino-Pearson test are used to test the normal distribution. Normal variables are expressed as average standard deviation, while non-normal variables are expressed as median and interquartile interval. Using parameter test ANOVA and Tukey back testing to compare normal distribution variables. For the data with non-normal distribution, Kruskal-Wallis test and Dunn back testing are used. Statistical test is used to evaluate whether there are statistically significant differences between groups. The difference is considered significant at the level of *p* < 0.05.

## Results

### Distribution of exogenous BMP-2 in orthodontic periodontal tissue

**FIGURE 3:**
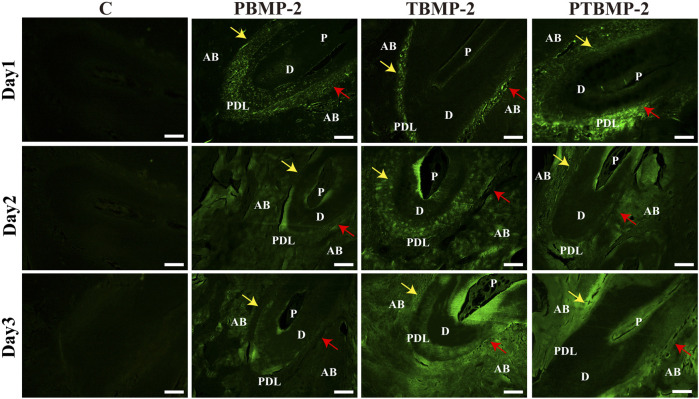
Distribution of exogenous BMP-2 in periodontal tissues of orthodontic tooth. The fluorescently labeled BMP-2 appeared green in color. Scale bar = 200 μm. AB, alveolar bone; D, dentin; PDL, periodontal ligament; P, dental pulp. Yellow arrows, pressure side of the root. Red arrows, tension side of the root. BMP-2 distribution could be seen in both pressure side and the tension side M1’s mesial root.

The sagittal section of the sample injected with fluorescently labeled BMP-2 shows the distribution of exogenous BMP-2 in the periodontal tissue of orthodontic teeth (Figure 3). Under confocal microscope, the fluorescently labeled BMP-2 was green. On the first day, the drug was mainly concentrated in periodontal tissue, less distributed in alveolar bone, and gradually spread to the surrounding alveolar bone on the second day. On the third day, the green fluorescent coloring drugs were mainly distributed in alveolar bone. Regardless of the injection site, BMP-2 was distributed in the proximal root of M1 on the pressure side and the tension side.

#### Tooth movement distance

**FIGURE 4:**
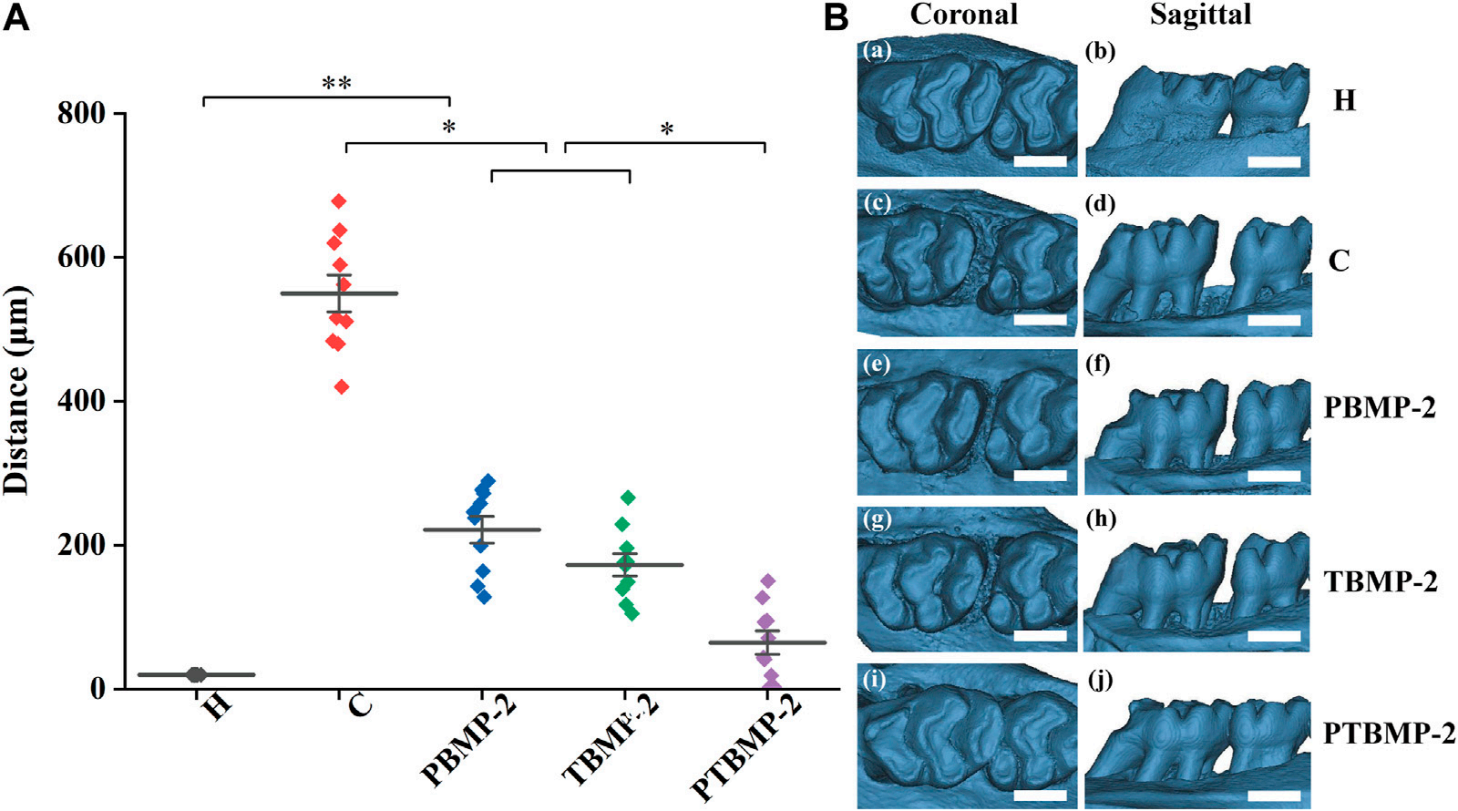
Mean and standard deviation of tooth movement (μm) measured by the horizontal distance between the highest convexity on the distal surface of first molars and the highest convexity on the mesial surface of second molars **(A)**. Representative three-dimensional constructive micro-CT views images showing tooth moving distance **(B)**, scale bar = 1,000 μm *Significant (*p* < 0.05) difference between groups, **Significant (*p* < 0.01) difference between groups.

By comparing the tooth movement of group C and group H (Figure 4), the OTM model was successfully established. The tooth movement distance of group H without orthodontic appliance was 20 μm. There was no statistical difference in tooth movement distance between PBMP-2 group and TBMP-2 group (*p* > 0.05), and the movement distance of both groups was significantly shorter than that of group C (*p* < 0.01). The tooth movement distance of PTBMP-2 group was significantly shorter than that of C group, PBMP-2 group and TBMP-2 group (*p* < 0.01). There was no statistical difference between PTBMP-2 group and H group (*p* > 0.05).

#### Bone reconstruction during OTM

**FIGURE 5:**
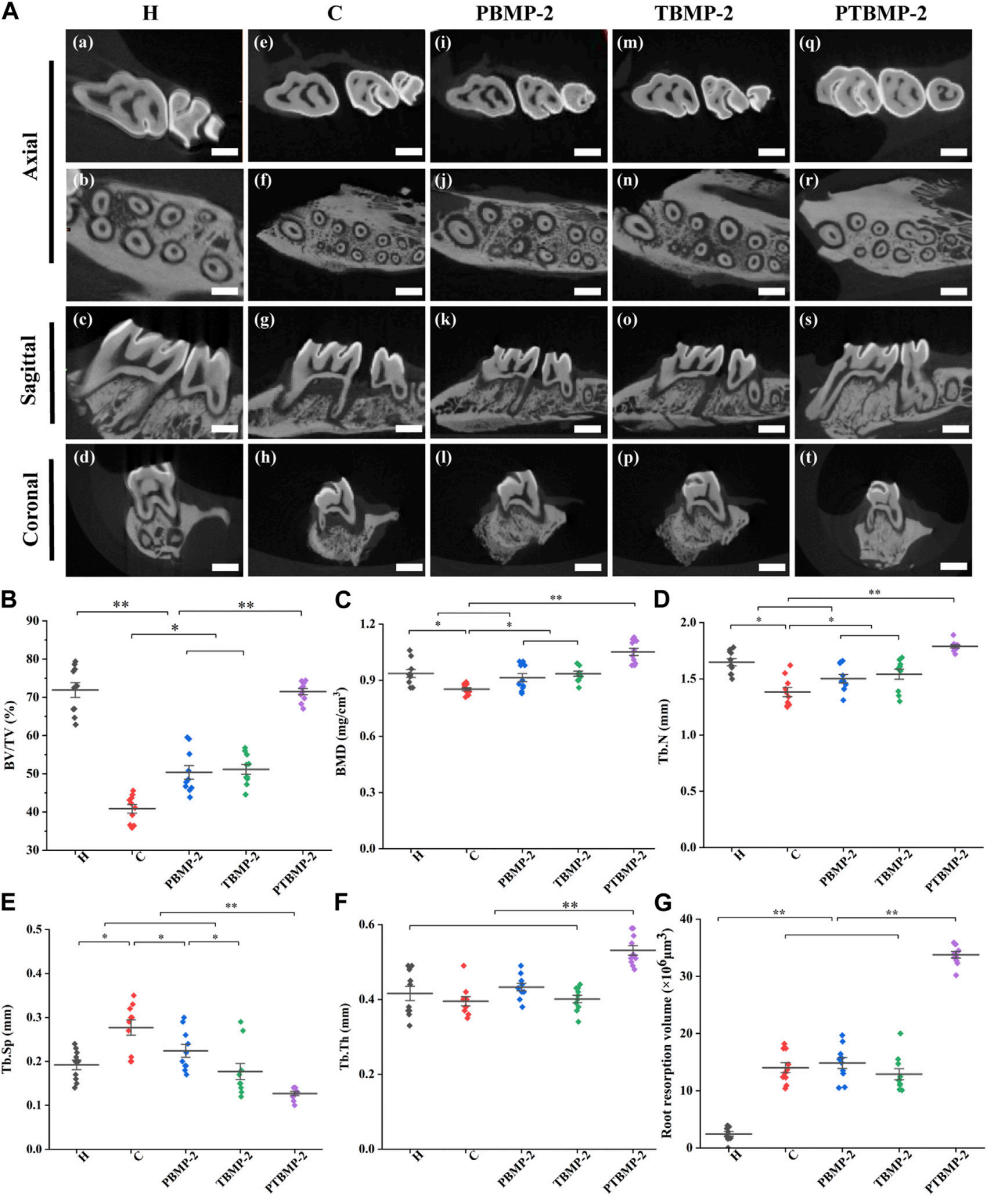
Bone remodeling observation in three dimensions of micro-CT, scale bar = 1000 μm **(A)**. Micro-CT measurement of the microarchitecture parameters in the selected ROI of the maxillary first molars: BV/TV **(B)**, BMD **(C)**, Tb.N **(D)**, Tb.Sp **(E)**, Tb.Th **(F)**. Root resorption volume of the mesial root of M1 cementum surface using Convex Hull algorithm **(G)**. Data are presented as mean and standard deviation. *Significant (*p* < 0.05) difference between groups, **Significant (*p* < 0.01) difference between groups.

The CT slice from three-dimensional direction reveals the qualitative change of microstructure parameter values in ROI (Figure 5A). The BV/TV of C, PBMP-2 and TBMP-2 groups was significantly lower than that of healthy control group and PTBMP-2 group (*p* < 0.01) (Figure 5B). There was no difference between PBMP-2 group and TBMP-2 group (*p* > 0.05), and the BV/TV ratio of both groups was significantly higher than that of group C (*p* < 0.05). In BMD (Figure 5C), there was no statistical difference between PBMP-2 group and TBMP-2 group (*p* > 0.05). Bone mineral density in both groups was significantly higher than that in group C (*p* < 0.05). PTBMP-2 group was significantly larger than the other four groups (*p* < 0.01). Trends in tuberculosis. N (Figure 5D) is consistent with BMD. Tuberculosis. Compared with other groups, the Sp in group C (Figure 5E) was significantly larger (*p* < 0.05). Tuberculosis. Sp in PBMP-2 group was higher than that in TBMP-2 group (*p* < 0.05). Tuberculosis. Sp in PTBMP-2 group was significantly lower than that in other groups (*p* < 0.01). Tuberculosis. Th (Figure 5F) in PTBMP-2 group was significantly higher than that in other four groups (*p* < 0.01).

#### Effect of OTM and BMP-2 local injection on root resorption

The results of micro-CT showed that the teeth receiving orthodontic traction showed different degrees of root resorption compared with group H (Figure 5G). After unilateral injection of bmp2, there was no significant difference in root resorption between the experimental group and the control group. The volume of root resorption pits in PTBMP-2 group was significantly larger than that in other groups (*p* < 0.01).

Morphological changes of periodontal tissue and quantitative determination of collagen fiber content during OTM.

**FIGURE 6:**
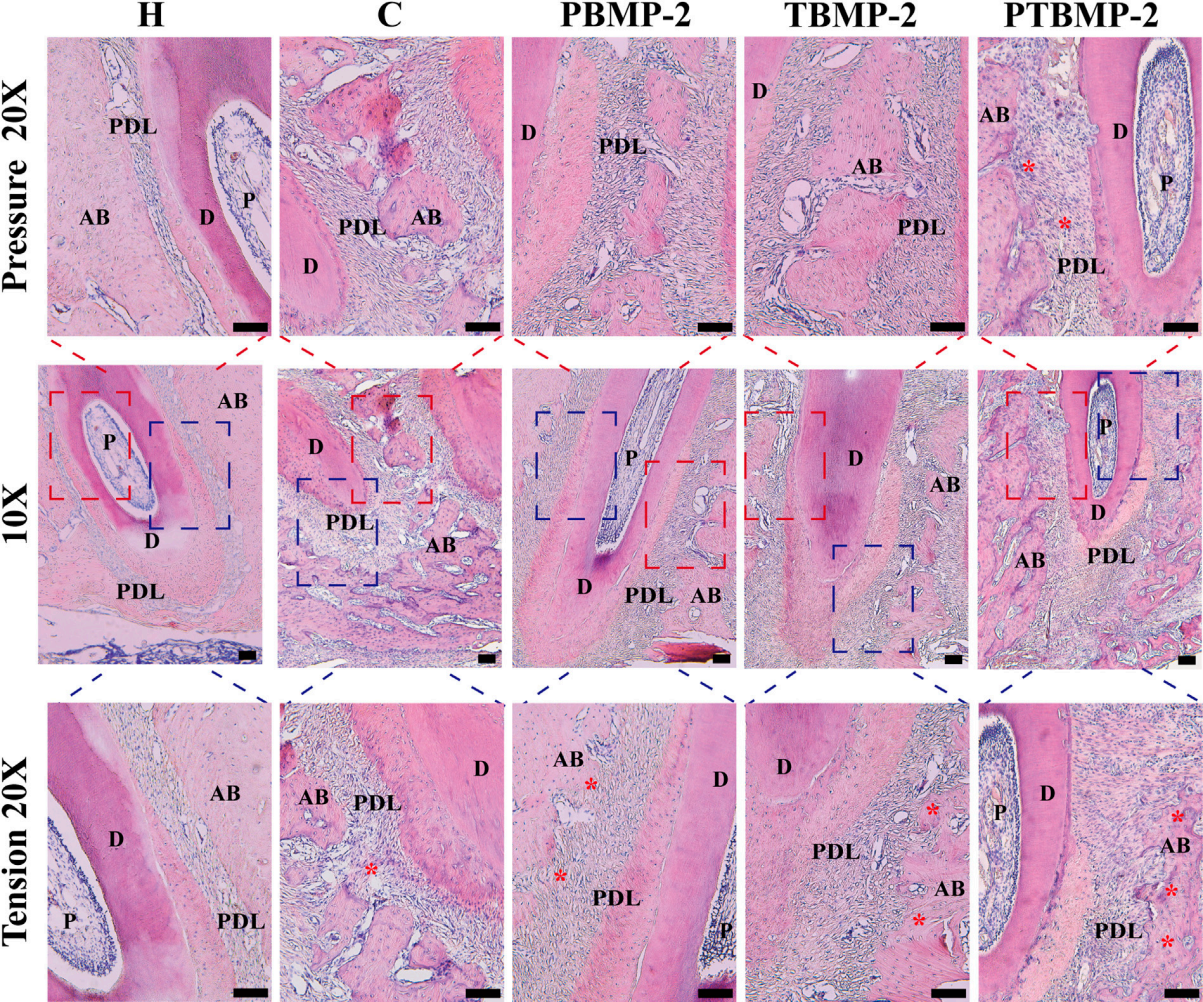
Light micrographs of sagittal hematoxylin-eosin (HE) stained sections of the mesial root of left maxillary first molar (M1) in ×10 magnification and ×20 magnification on the pressure and tension sides. Red boxes, periodontal tissue of pressure side; blue boxes, periodontal tissue of tension side. Scale bar = 100 μm. AB, alveolar bone; D, dentin; PDL, periodontal ligament; P, dental pulp. *, new bone deposition.

On HE stained sections, it was noted that the periodontal fiber arrangement and gap width in group H were consistent (Figure 6 a–c). With the traction of orthodontic force, the mesial root of M1 periodontal space on the pressure side is thinner than that on the tension side, showing the performance of pressure. On the pressure side, the alveolar bone surface is irregular and continuous, and shows positive absorption. In addition, periodontal fibers are disordered and broken (discontinuous), and a wide gap can be seen in the section. Compared with the pressure side, the periodontal ligament tissue is wider, the periodontal fibers seem to be stretched, and the fibers are longer and arranged more orderly (Figure 6 d–o). At the same time, new bone deposition and bone formation were found on the tension side of alveolar bone surface (Figure 6 f, i, l, o). On the tension side and pressure side of PTBMP-2 group, there were significant new bone deposits (Figure 6 m, o).

**FIGURE 7:**
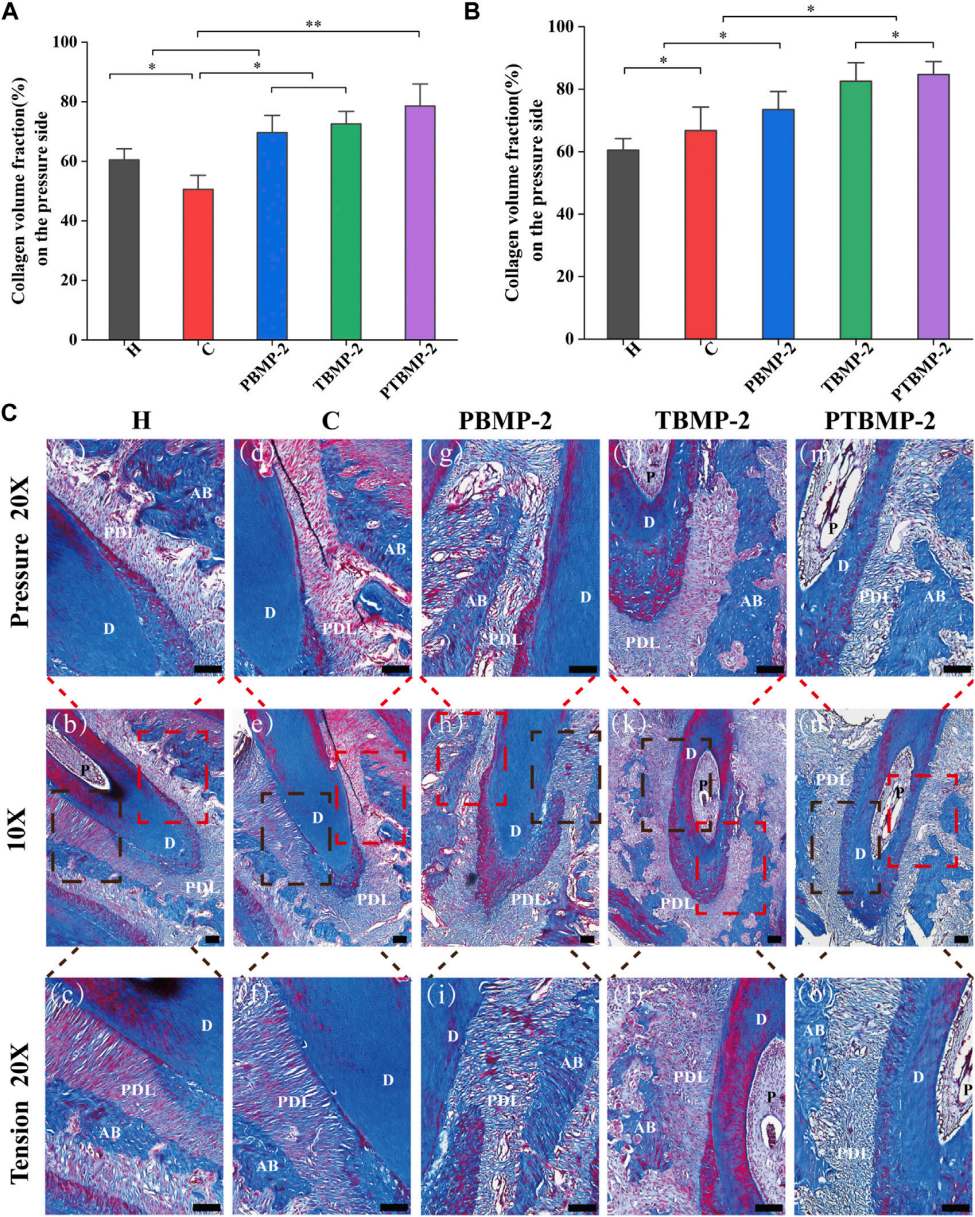
Histological measurement of collagen fiber content on Masson-stained sections. The periodontal ligament was measured on the pressure **(A)** and tension **(B)** sides of the mesial root of M1. The collagen volume fraction (CVF) of each side was measured on three standard fields and averaged. Data are presented as mean and standard deviation. *Significant (*p* < 0.05) difference between groups, **Significant (*p* < 0.01) difference between groups. Light micrographs of sagittal Masson-stained sections of the mesial root of left maxillary first molar (M1) in ×10 magnification and ×20 magnification on the pressure and tension sides **(C)**. Red boxes, periodontal tissue of pressure side; black boxes, periodontal tissue of tension side. Scale bar = 100 μm. AB, alveolar bone; D, dentin; PDL, periodontal ligament.

Masson staining section showed that the collagen fibers in group H were arranged neatly, without breakage, and the staining was even (Figure 7C a–c). In the experimental group receiving orthodontic traction, the collagen fiber bundle on the pressure side of the root became narrow, partially broken into strips and disorganized, and the area percentage of blue collagen fiber was lower than that on the tension side (Figure 7C d–o). In the experimental group injected with BMP-2, compared with group C and group H, the percentage of collagen on the pressure side and tension side is larger.

Collagen volume fraction (CVF) was calculated after semi-quantitative measurement and analysis of collagen content in the pressure side and tension side of the first molar. The results showed that on the pressure side (Figure 7B), the CVF of BMP-2 injection group increased compared with H and C groups, while that of blank control group decreased compared with H group (*p* < 0.05). In tension (Figure 7A), compared with group H, the four experimental groups have significantly increased (*p* < 0.05). There was no difference in CVF between T-BMP and PTBMP-2 groups (*p* > 0.05), but it was significantly increased compared with other groups (*p* < 0.05).

### Effects of OTM and BMP-2 injection on the formation of osteoclasts and odontoblasts

**FIGURE 8:**
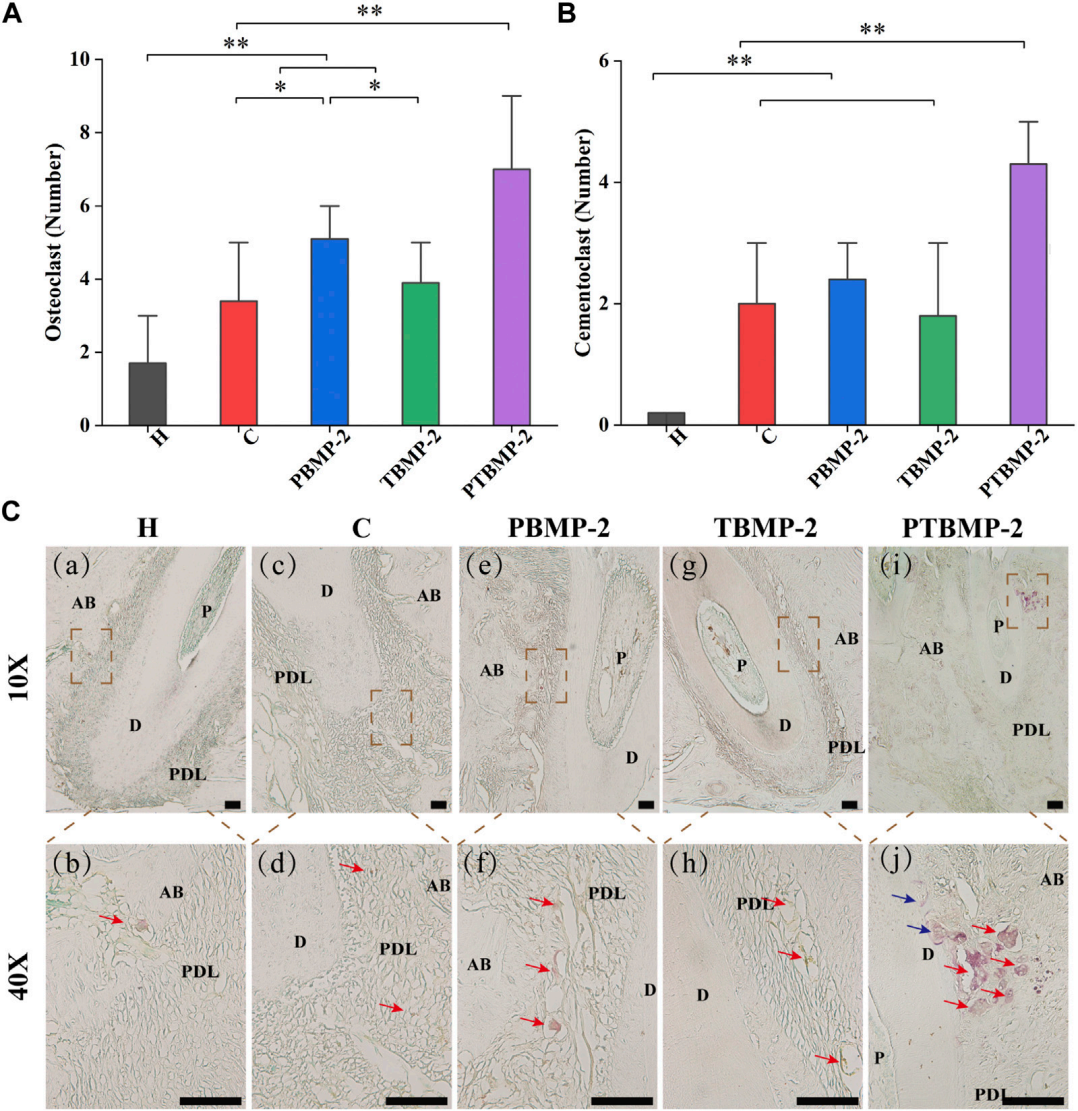
Histological measurement of osteoclasts **(A)** and cementoclasts **(B)** on TRAP-stained sections. Data are presented as mean and standard deviation. *Significant (*p* < 0.05) difference between groups, **Significant (*p* < 0.01) difference between groups. Light micrographs of sagittal TRAP-stained sections of the mesial root of left maxillary first molar (M1) in ×10 magnification and ×40 magnification **(C)**. →, osteoclast appeared as trap-stained red-positive multinucleated giant cells found in resorption traps or on the surface of the alveolar bone. →, cementoclasts appeared as positive multinucleated giant cells on the root surface deep within the resorption traps of dentin. Scale bar = 100 μm. AB, alveolar bone; D, dentin; PDL, periodontal ligament.

On the section stained by TRAP, cementum-destroying cells showed positive multinucleated giant cells on the root surface deep in the dentin absorption trap, while osteoclasts showed red multinucleated giant cells stained by trap in the absorption trap or on the alveolar bone surface (Figure 8C). The results of Trap positive cell count showed that osteoclasts in PBMP2 group were significantly more than those in C group and TBMP-2 group (Figures 8A, C). The number of cemental destruction cells in dental slices with orthodontic augmentation devices is much higher than that in group H (Figures 8B, C). Compared with the other four groups, the number of osteoclasts and cementum-destroying cells in PTBMP-2 group increased significantly.

#### Biosafety of BMP-2 local injection *in vivo*

The H&E staining results of the vital organs of rats (including heart, liver, spleen, lung and kidney sections) are shown in Supplementary Files, and no abnormality or pathological morphology is observed in all tissue sections, which indicates that there is no abnormality in the functions of the vital organs of experimental animals. And it is reasonable to expect that injecting a certain dose of BMP-2 into periodontal tissue has good biological safety *in vivo*.

## Discussion

The regulation of OTM has been popularized in orthodontic research (Yamaguchi et al., 2010). Tooth movement in response to mechanical stress is a complex process (Will, 2016; Shoji-Matsunaga et al., 2017; Odagaki et al., 2018). According to the significant statistical differences in tooth movement distance, microstructure parameters and histological manifestations between BMP-2 injection group and blank control group, local application of BMP-2 around orthodontic teeth can reduce movement distance, increase collagen fiber content and enhance tooth stability. According to the difference between unilateral and bilateral injection groups, this enhancement is dose-dependent, and the distribution of BMP-2 labeled by fluorescence is position-independent. 0.5 μg/mL BMP-2 of 60 μL per tooth may be used as a drug to control orthodontic tooth movement without increasing the risk of root resorption and organ damage.

In the family of bone morphogenetic proteins (BMPs), BMP-2 can activate the formation of osteoblasts and osteoclasts, which may regulate the rate of bone remodeling (Simonet et al., 1997; Kaneko et al., 2000; Kamiya et al., 2008a; Kamiya et al., 2008b). The changes of alveolar bone provide biological basis for the designed tooth movement. The application of orthodontic force initiates the cellular and molecular activities that lead to periodontal remodeling. In the periodontal ligament area of stressed teeth, tension side and pressure side are generated during orthodontic treatment of tooth movement. Tension causes periodontal tissue to stretch, and osteoblasts (OB), alkaline phosphate (ALP), type I collagen and bone morphogenetic protein (BMPs) are gradually induced, which promotes bone formation and tissue remodeling (Wescott et al., 2007). At the same time, on the pressure side, periodontal tissue is compressed, which leads to the activation of osteoclasts and the absorption of alveolar bone. Finally, the dynamic balance is achieved, in which the bone absorption in the traction direction is dominant, which leads to the teeth moving in response to orthodontic force. If we can better understand the complex function of BMP-2 in controlling chondrocytes, osteoblasts and osteoclasts, it will be beneficial to the development of therapeutic methods. Considering the importance of experimental rat model in clarifying biological mechanism and finding new treatment methods in orthodontic field, we designed this *in vivo* study to study the effects of local application of BMP-2 in different parts of periodontal tissue on tooth movement and periodontal tissue remodeling.

According to the experimental rat model, orthodontic teeth move fastest between 7 and 14 days, and then slow down between 14 and 21 days (Ru et al., 2013). Based on the results of tooth movement distance within 16 days, it was found that the movement of orthodontic teeth with local injection of BMP-2 was limited compared with those without injection. As shown by the changes of microscopic parameters on micro-CT, BV/TV, BMD and Tb in BMP-2 injection group showed an upward trend. The performance of PBMP-2 and TbMP-2 groups is similar, except TB. The Sp index of PBMP-2 group was higher. Trabecular bone is an irregular network three-dimensional structure, which is an inward extension of bone cortex. It is used to store calcium and maintain bone elasticity and strength. The study on the microscopic characteristics of trabecula during orthodontic tooth movement can be used to understand the effects of various intervention measures (Gokce et al., 2008). During orthodontic tooth movement, BV/TV, Tb and other micro-parameters. Th decreased significantly on the pressure side and increased on the tension side (Ru et al., 2013). Compared with group H, this established model is obvious in group C. Applying clinically relevant amount of BMP-2 around mechanically loaded teeth will increase bone growth, thus reducing orthodontic traction resistance.

When comparing PBMP-2 and TBMP-2 groups, the injection site did not affect the clinical manifestations. Drugs injected on the tension side of periodontal ligament can be delivered to the pressure side, because periodontal ligament is a single compartment that can communicate. There are no strict definitions of pressure surface and tension surface, except for the academic term (Iglesias-Linares et al., 2012). Therefore, the injection site has nothing to do with the clinical effect of locally delivered drugs on tooth movement. In this study, the distribution of fluorescently labeled BMP-2 on the slice also confirmed this theory. The application of BMP-2 on the tension side will not stimulate tooth movement, which supports the hypothesis that the osteoclast activity and bone resorption activity on the pressure side determine the OTM speed (Li et al., 2021). At the same time, the fluorescently labeled sections showed that there was fluorescence aggregation in the dentinal tubule adjacent to the pulp, indicating that the drugs injected during periodontal period may spread along the dentinal tubule.

The results of osteoclast count showed that compared with group C, osteoclasts in PBMP-2 and TBMP-2 injection groups were activated, but bone absorption was not dominant. Collagen fibers in periodontal tissue play an important role in the changes of periodontal tissue and alveolar bone during tooth movement. The increase of type I collagen is beneficial to bone synthesis and tissue remodeling (Wescott et al., 2007). The morphological observation and semi-quantitative results of collagen content in this study confirmed the histological changes of collagen during tooth movement, and it was also found that local injection of BMP-2 into periodontal tissue increased the collagen content around orthodontic teeth (Anastasi et al., 2008).

Anchorage is one of the most challenging concepts in orthodontics. In order to achieve the desired orthodontic treatment goal, the anchorage teeth must be in a stable position to resist the back pressure exerted by the target teeth. The displacement of anchorage teeth may prolong the treatment time and reduce the clinical effect. It is reported that there are several ways to enhance anchorage, including intraoral and extraoral devices, micro-screws and drugs (Borsos et al., 2012; Fernández-González et al., 2016). Additional devices may be invasive and increase patient discomfort. Recent studies have focused more on drug therapy to directly or indirectly inhibit bone resorption and avoid unwanted tooth movement (Hudson et al., 2012; Yabumoto et al., 2013). There is no difference in the movement distance between the double dose PTBMP-2 group and the H group, indicating that there is no significant movement under 30 g traction. There were significant differences in tooth displacement and trabecular microscopic parameters between PTBMP-2 group and unilateral injection group. Compared with single injection, double injection of BMP-2 in periodontal tissue showed stronger osteogenic activity and anti-traction ability. The current results show that the osteogenic function of BMP-2 is dose-dependent to some extent. At the same time, Miyaji et al. (2006) found that BMP-2 could lead to a dose-dependent increase of osteoclasts’ dentin absorption at an *in vitro* dose of 400 mg/mL.

As a common complication of orthodontic tooth movement, root resorption may occur up to 100% of the time and should be avoided as much as possible (Makrygiannakis et al., 2019). Severe root resorption will lead to imbalance of crown-root ratio, tooth loosening or even falling off, and other adverse health effects. Gonzales et al. (2008) found that different orthodontic forces may lead to root resorption during orthodontic tooth movement. The root resorption increases with time and eventually tends to be stable. Excessive orthodontic force causes the root resorption to increase. On the third day after the application of force, active osteoclasts and odontoblasts could be observed. On the seventh day, the absorption of root and alveolar bone was the most obvious, and the most obvious change occurred in the top 1/3 of the pressure side (Verna et al., 1999; Tomizuka et al., 2007). There was no significant difference in root resorption volume between unilateral BMP-2 injection group and group C, indicating that proper injection of BMP-2 into periodontal tissue of orthodontic teeth did not promote root resorption. On the contrary, compared with other experimental groups, bilateral BMP-2 injection leads to high local tissue concentration of drugs, stimulates cementum destruction activity, and leads to significant root absorption. As a part of the current research, we use the mature method developed by Harris et al. (2006) to calculate the volume of root absorption pit by using Micro-CT root model and convex Hell algorithm. Micro-CT can analyze tooth movement, three-dimensional structure of root surface and microstructure parameters of alveolar bone without destroying the integrity of samples, and provide enhanced accuracy, reproducibility and technical sensitivity. Different from the reconstruction model in traditional methods (Chan and Darendeliler, 2004; Chan et al., 2004), the three-dimensional root model obtained by micro-CT is real, not simulated by computer, which leads to more accurate and real measurement.

It is a promising clinical application strategy to combine bone homeostasis regulator with innovative drug delivery system or program. According to the research results, periodontal application of BMP-2 seems to be a potential treatment method, which can accurately correct tooth movement without increasing root resorption. However, too high local concentration of BMP-2 may lead to root resorption on the pressure side of the root and should be avoided. Care must be taken to prevent drug accumulation. However, this study has potential limitations. It needs a site-specific drug delivery method with long-term effect to determine the best regulator with maximum effectiveness and minimum side effects.

## Conclusion

This *in vivo* study shows that local application of BMP-2 around orthodontic teeth can enhance bone mass and tooth anchorage and help reduce tooth movement. Injecting BMP-2 into periodontal tissue restricts the movement of teeth, rather than accelerating the movement of orthodontic teeth. When administered around orthodontic teeth in a clinical amount, osteogenesis effects of BMP-2 were dose-dependent and site-independent. High levels of BMP-2 in the periodontal tissue of orthodontic teeth may activate osteoclasts and cementoclasts, causing aggressive root resorption. Therefore, we believe local application of BMP-2 has the potential to precisely regulate orthodontic tooth movement and periodontal tissue remodeling with *in vivo* biosafety.

## Data Availability

The original contributions presented in the study are included in the article/Supplementary Material, further inquiries can be directed to the corresponding authors.

## Abbreviations

ALP, alkaline phosphate; BMP-2, bone morphogenetic protein-2; BMD, bone mineral density; BMPs, bone morphogenetic proteins; CT, computed tomography; CVF, collagen volume fraction; HE, Hematoxylin-eosin; OTM, Orthodontic tooth movement; OPG, osteoprotegerin; OC, osteoclasts; PDL, periodontal ligament; PAOO, periodontally accelerated osteogenic orthodontics; PBMP-2, 2-Pressure BMP-2; PTBMP-2, 4-Pressure and Tension BMP-2; ROI, region of interest; TBMP-2, 3-Tension BMP-2; TRAP, Tartrate-resistant acid phosphatase.
