# Supplementary figures and images for: Effect of local application of bone morphogenetic protein -2 on experimental tooth movement and biological remodeling in rats
Source: Front Physiol. 2023 Apr 18;14:1111857. doi: 10.3389/fphys.2023.1111857 (PMC10151543; doi:10.3389/fphys.2023.1111857)

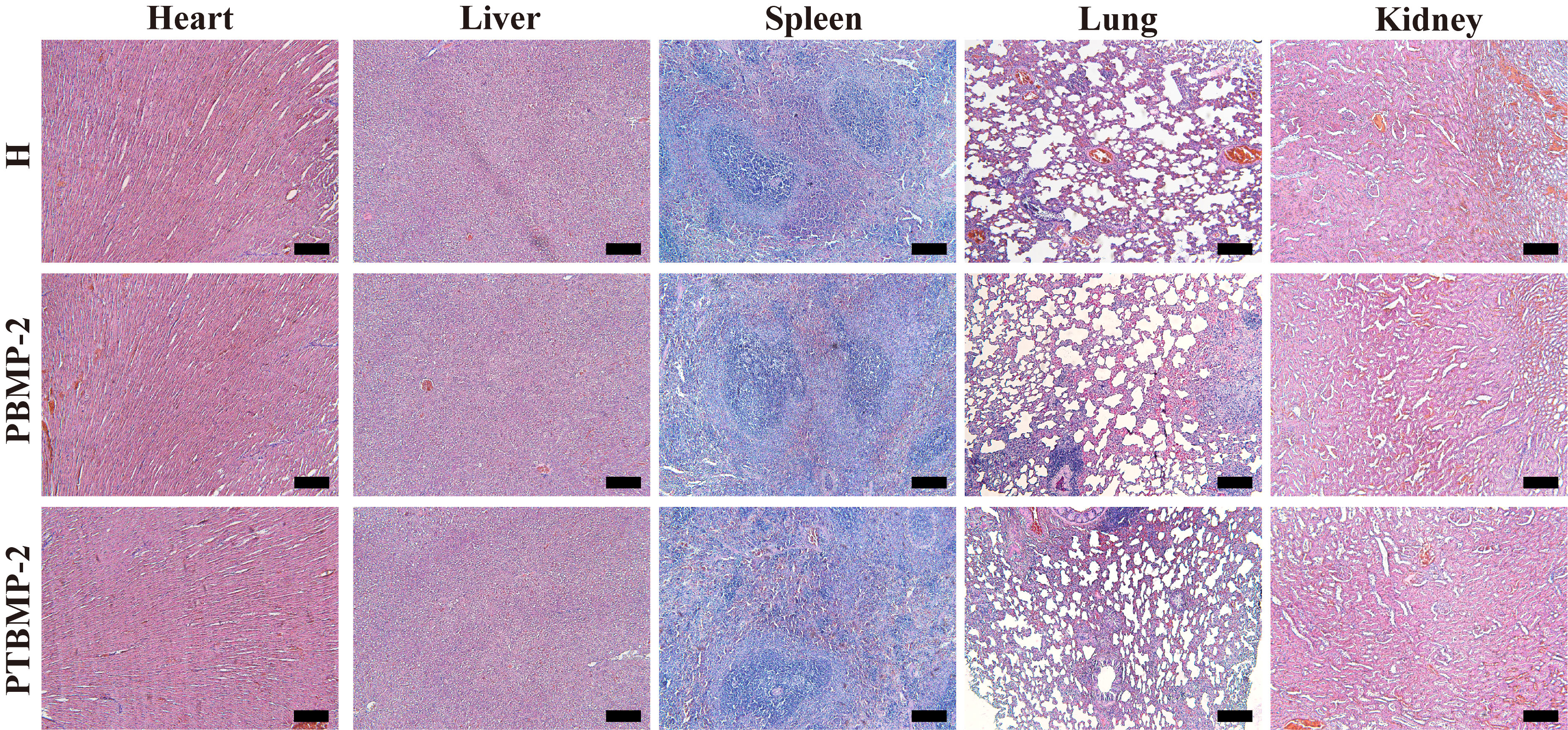

Supplement: Supplementary file 1 [file Image1.jpg]
